# Liraglutide Improves Pancreatic Beta Cell Mass and Function in Alloxan-Induced Diabetic Mice

**DOI:** 10.1371/journal.pone.0126003

**Published:** 2015-05-04

**Authors:** Kanako Tamura, Kohtaro Minami, Maya Kudo, Keisuke Iemoto, Harumi Takahashi, Susumu Seino

**Affiliations:** Division of Cellular and Molecular Medicine, Kobe University Graduate School of Medicine, Kobe, Japan; Universidad Miguel Hernández de Elche, SPAIN

## Abstract

Glucagon-like peptide-1 (GLP-1) receptor agonists potentiate glucose-induced insulin secretion. In addition, they have been reported to increase pancreatic beta cell mass in diabetic rodents. However, the precise mode of action of GLP-1 receptor agonists still needs to be elucidated. Here we clarify the effects of the human GLP-1 analog liraglutide on beta cell fate and function by using an inducible Cre/loxP-based pancreatic beta cell tracing system and alloxan-induced diabetic mice. Liraglutide was subcutaneously administered once daily for 30 days. The changes in beta cell mass were examined as well as glucose tolerance and insulin secretion. We found that chronic liraglutide treatment improved glucose tolerance and insulin response to oral glucose load. Thirty-day treatment with liraglutide resulted in a 2-fold higher mass of pancreatic beta cells than that in vehicle group. Liraglutide increased proliferation rate of pancreatic beta cells and prevented beta cells from apoptotic cells death. However, the relative abundance of YFP-labeled beta cells to total beta cells was no different before and after liraglutide treatment, suggesting no or little contribution of neogenesis to the increase in beta cell mass. Liraglutide reduced oxidative stress in pancreatic islet cells of alloxan-induced diabetic mice. Furthermore, the beneficial effects of liraglutide in these mice were maintained two weeks after drug withdrawal. In conclusion, chronic liraglutide treatment improves hyperglycemia by ameliorating beta cell mass and function in alloxan-induced diabetic mice.

## Introduction

Glucagon-like peptide-1 (GLP-1), an incretin, is released from enteroendocrine L-cells in response to ingested nutrients in the lumen of the gut [1]. GLP-1 stimulates insulin secretion in a glucose concentration-dependent manner, and inhibits glucagon secretion through its specific receptor [2, 3]. Because its action is glucose-dependent, GLP-1 has low risk of hypoglycemia [1]. In addition, GLP-1 slows gastric emptying [4] and reduces appetite [5], which have favorable effects in controlling glycemia. However, native GLP-1 exhibits a very short half-life in plasma due to degradation by dipeptidyl peptidase-IV (DPP-IV), which compromises its therapeutic potential [6]. To overcome the problem of the short duration of GLP-1 action, GLP-1 receptor agonists resistant to DPP-IV have been developed [7]. Exenatide is a synthetic form of exendin-4, an incretin mimetic originally isolated from saliva of the Gila monster having a function similar to GLP-1 [8]. Liraglutide, another GLP-1 receptor agonist, is a long-acting human GLP-1 analogue with an amino acid substitution and a fatty acid side chain designed for protection from degradation by DPP-IV [9]. Both drugs are now used clinically worldwide.

Exenatide was reported to normalize both first and second phases of glucose-induced insulin secretion in patients with type 2 diabetes during 3-hour intravenous infusion [10] and to provide long-term improvement of beta cell function by chronic treatment [11]. Liraglutide also increases both first and second phases of insulin secretion in type 2 diabetic patients [12]. The LEAD (liraglutide effect and action in diabetes) studies revealed that chronic treatment of type 2 diabetes with liraglutide significantly improved beta cell function as evaluated by homeostasis model assessment of pancreatic beta cell function (HOMA-beta) [7, 13].

Chronic treatment with GLP-1 receptor agonists has also been shown to increase pancreatic beta cell mass in diabetic rodents [14]. Beta cell mass is thought to be determined by a balance among cell proliferation, cell death, and neogenesis. Experimental evidence indicates that GLP-1 receptor agonists increase beta cell proliferation and decrease beta cell apoptosis [14]. However, the effect of GLP-1 receptor agonists on beta cell neogenesis is controversial, mainly due to the lack of methodology for assessing neogenesis. Dor et al developed a method using genetically modified mice in which insulin-expressing cells can be specifically labeled at desired time points to allow discrimination of proliferation and neogenesis of beta cells by tracing labeled cells [15]. By applying a similar methodology, we established Ins2-CreERT2/R26R-YFP double knock-in mouse model and found that pancreatic beta cells are generated not only by proliferation of pre-existing beta cells but also by neogenesis from non-beta cells even after birth [16].

In the present study, a diabetic mouse model induced by alloxan is used to evaluate the effects of liraglutide on beta cell mass and function. Alloxan generates reactive oxygen species (ROS) in pancreatic beta cells and damages the cells [17], as is seen in patients with type 2 diabetes [18]. Thus, the alloxan-induced diabetic mouse is a model of beta cell failure caused by ROS. We utilize Ins2-CreERT2/R26R-YFP mice to trace beta cells to determine whether liraglutide induces neogenesis of beta cells. We also examine changes in beta cell function during liraglutide treatment and after withdrawal of the drug.

## Materials and Methods

### Animals

**Fig 1:**
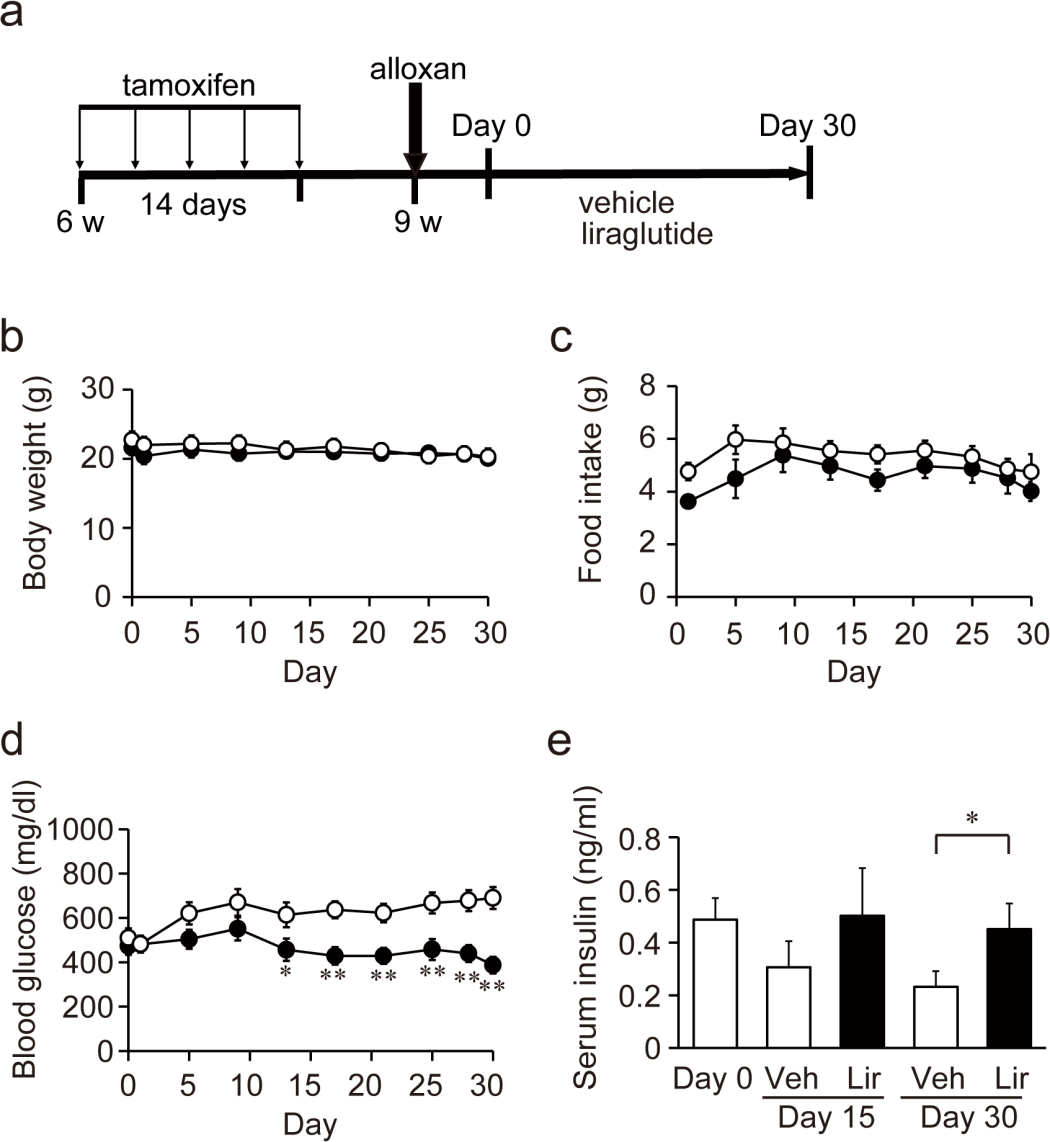
Metabolic parameters in alloxan-diabetic mice treated with vehicle or liraglutide for 30 days. (**a**) Schematic representation of the study. (**b**) Body weight. There is no difference between two groups. (**c**) Food intake. Liraglutide slightly decreased food intake compared with vehicle. (**d**) Blood glucose. Blood glucose levels were significantly lower in liraglutide-treated group than in vehicle-treated group after day 13. (**e**) Serum insulin. Serum insulin levels were significantly higher in liraglutide-treated group than in vehicle-treated group on day 30. White circles and bars represent vehicle-treated group (Veh) (n = 9–13), and black circles and bars represent liraglutide-treated group (Lir) (n = 10–15). *p < 0.05, **p < 0.01

Ins2-CreERT2/R26R-YFP double knock-in mice, in which the pancreatic beta cells can be labeled specifically and permanently upon injection of tamoxifen, were generated as described previously [16, 19]. All mice had free access to food and water and were kept on a 12 h light, 12 h dark cycle. For labeling and tracing of pancreatic beta cells, male Ins2-CreERT2/R26R-YFP mice at 6 weeks of age were injected intraperitoneally with tamoxifen (Sigma-Aldrich, St Louis, MO) five times (4 mg/head) within 2 weeks. Ten days after the last injection of tamoxifen, alloxan (Sigma-Aldrich) was administered intraperitoneally to the mice at 60 mg/kg body weight. On the next day (day 0), mice with blood glucose concentration above 300 mg/dl were used for the study (Fig 1A). Animal care and experimental procedures were approved by the Animal Ethics Committee of Kobe University Graduate School of Medicine (Permit Number: 23-06-09) and carried out according to the Kobe University Experimentation Regulations. All surgery was performed under sodium pentobarbital anesthesia, and all efforts were made to minimize suffering.

### Drug treatment

Liraglutide (200 μg/kg body weight, Novo Nordisk, Bagsvaerd, Denmark) or vehicle (physiological saline) was administered subcutaneously to the tamoxifen-injected, alloxan-treated Ins2-CreERT2/R26R-YFP mice once daily for 30 days.

### Metabolic parameters

Body weight, food intake, blood glucose levels, and serum insulin levels were routinely measured. Blood samples were obtained from tail vein in the morning before drug administration, and sera were stored at -80°C until use. Blood glucose concentrations were measured by Antsense III glucose analyzer (Bayer Yakuhin, Osaka, Japan). Serum insulin levels were determined by an ELISA kit (Morinaga, Yokohama, Japan).

### Glucose tolerance test

Oral glucose tolerance tests (OGTT) were performed in mice fasted 16 hours. Glucose (1.5 g/kg body weight) was administered orally 24 hours after last liraglutide administrations, and blood samples were obtained from tail vein. Blood glucose and serum insulin levels were measured as described above. Insulinogenic index was calculated as the ratio of the change in insulin (μU/ml) and glucose (mg/dl) responses from 0 to 15 minutes.

### Immunohistochemistry

Pancreata removed from the mice were fixed with 10% formalin and embedded in paraffin. The 5 μm sections of embedded tissue were incubated overnight at 4°C with primary antibodies: guinea pig polyclonal anti-insulin (ab7842, Abcam, Tokyo, Japan) (1:100), mouse monoclonal anti-glucagon (G2654, Dako Japan, Tokyo, Japan) (1:2,000), rabbit monoclonal anti-GFP (A-11122, Molecular Probes, Eugene, OR) (1:200), rat monoclonal anti-mouse Ki67 (M7249, Dako Japan) (1:500), rabbit polyclonal anti-Pdx1 (07–696, Millipore, Billerica, MA, USA) (1:500), mouse monoclonal anti-FoxO1 (05–1075, Millipore) (1:100), or mouse monoclonal anti-4-hydroxy-2-noneal modified protein (4-HNE) (MHN-100P, Japan Institute for the Control of Aging, Shizuoka, Japan) (25 μg/ml) [20]. For Pdx1 staining, antigen retrieval was performed by autoclave heating in 10 mM citrate buffer (pH 6.0) before primary antibody incubation. The sections were then incubated with secondary antibodies: Alexa Fluor 488–conjugated goat anti-guinea pig IgG (A-11073, Molecular Probes) (1:500), Alexa Fluor 546–conjugated goat anti-mouse IgG (A-11030, Molecular Probes) (1:500), Alexa Fluor 546–conjugated goat anti-rat IgG (A-11081, Molecular Probes) (1:500), or Alexa Fluor 546–conjugated goat anti-rabbit IgG (A-11035, Molecular Probes) (1:500) in a dark chamber for 1.5 h at room temperature for detection. Biotinylated DBA lectin (B-1035, Vector Labs, Burlingame, CA) (1:200) and Alexa Fluor 546-conjugated streptavidin (S-11225, Molecular Probes) (1:500) were used to identify duct cells. Nuclei were visualized by DAPI (Dojindo, Kumamoto, Japan) (1:2,000). The stained sections were observed with BZ9000 microscope (Keyence, Osaka, Japan).

### Quantification of images

For examining neogenesis, frequency of YFP-labeled beta cells (labeling index) was calculated by dividing the number of YFP/insulin double-positive cells by the number of total insulin-positive cells [16]. For measurement of beta cell or alpha cell mass, we first measured percent area of insulin-positive cells or glucagon-positive cells in the pancreas from five distinct sections of each mouse pancreas. The beta cell mass or the alpha cell mass was then estimated by multiplying the insulin-positive cell area or the glucagon-positive cell area, respectively, by the pancreas weight of corresponding animals [16]. Apoptosis was quantified by TUNEL method using In Situ Cell Death Detection Kit (Roche Applied Science, Tokyo, Japan).

### Statistical analysis

Data are presented as means ± SEM. The significance of difference between test groups was evaluated by use of student *t*-test or multiple analysis of Tukey-Kramer’s test. *p* < 0.05 was considered significant.

## Results

### Liraglutide ameliorates blood glucose and insulin levels

We measured body weight, food intake, blood glucose and serum insulin levels in alloxan-induced diabetic Ins2-CreER/R26R-YFP mice. Alloxan treatment blocked body weight gain throughout the experimental period (30 days), and liraglutide did not affect body weight (Fig 1B). Food intake was slightly decreased in mice with liraglutide treatment compared to that in control mice (Fig 1C). The mice had blood glucose levels as high as 400–500 mg/dl one day after alloxan-treatment and the levels gradually increased in the vehicle group (Fig 1D). Daily liraglutide injection prevented the rise in blood glucose levels; the difference in levels between vehicle and liraglutide groups was statistically significant after day 13 (Fig 1D). In accord with the improved hyperglycemia, liraglutide-treated mice showed significantly higher serum insulin levels than vehicle-treated mice on day 30 (Fig 1E).

### Liraglutide improves glucose tolerance with recovered insulin response

**Fig 2:**
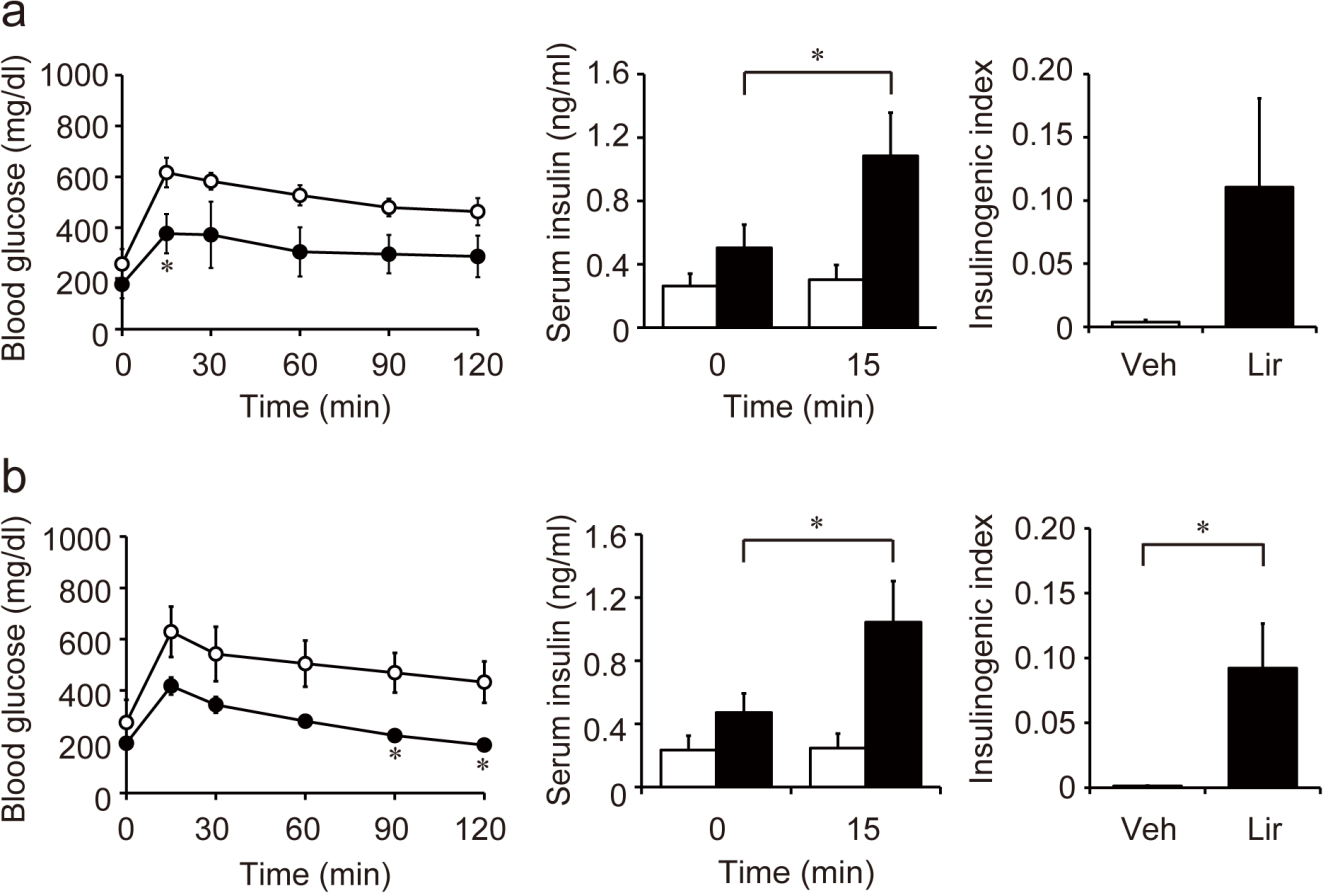
Oral glucose tolerance test (OGTT). Blood glucose and serum insulin levels during 1.5 g/kg OGTT on day 15 (**a**) and day 30 (**b**) are shown. Glucose tolerance was significantly improved by liraglutide treatment. Although insulin response was not detected in vehicle treated group, liraglutide improved the response. Insulinogenic index was significantly higher in liraglutide-group than vehicle-group on day 30. White circles and bars represent vehicle-treated group (Veh) (n = 5–6), and black circles and bars represent liraglutide-treated group (Lir) (n = 5–6). *p < 0.05

Alloxan injection lead to glucose intolerance concomitant with fasting hyperglycemia and almost complete loss of insulin response (S1 Fig). Daily liraglutide injection significantly suppressed blood glucose levels after oral glucose challenge (Fig 2). Serum insulin was clearly increased by glucose load in the liraglutide-treated group but not in the vehicle-treated group both on day 15 (Fig 2A) and day 30 (Fig 2B). These results suggest that liraglutide improves glucose tolerance in alloxan-induced diabetic mice by restoring beta cell insulin secretory function.

### Liraglutide prevents loss of beta cell mass

**Fig 3:**
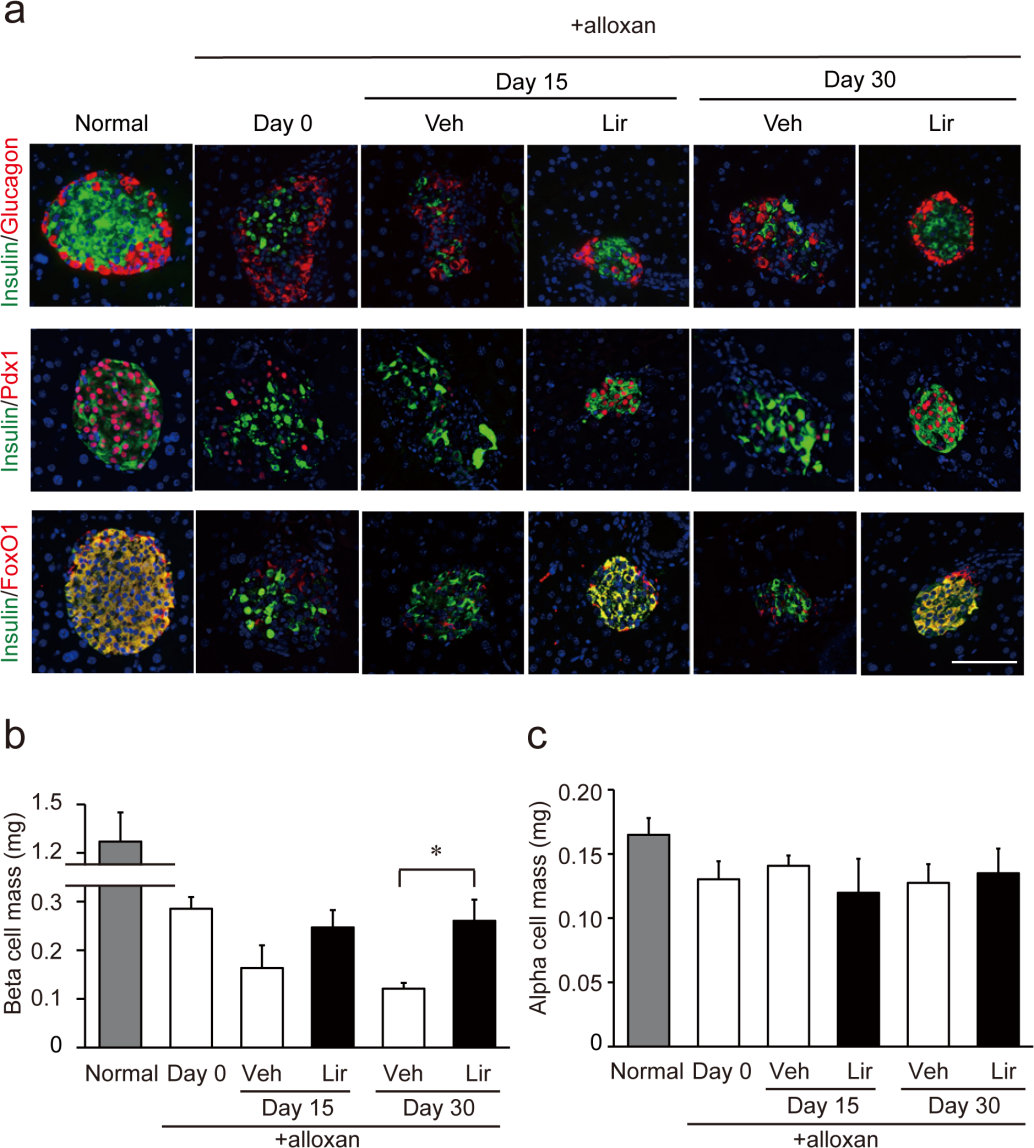
Pancreatic beta cell mass and alpha cell mass. (**a**) Insulin/glucagon (upper), insulin/Pdx1 (middle), and insulin/FoxO1 (bottom) double immunostaining. Representative images of islets are shown. Scale bars, 100 μm. (**b**) Beta cell mass. Alloxan treatment markedly decreased beta cell mass. The beta cell mass was two-fold higher in liraglutide-treated mice than that in vehicle-treated mice on day 30. (**c**) Alpha cell mass. Alpha cell mass did not change by alloxan treatment. Liraglutide did not affect alpha cell mass. White bars represent vehicle-treated group (Veh) (n = 4–9), and black bars represent liraglutide-treated group (Lir) (n = 4–9). *p < 0.05

Immunohistochemical analysis showed that alloxan treatment immediately caused severe loss of pancreatic beta cells (insulin-positive cells) and islet structure was gradually destroyed (Fig 3A). We found that liraglutide treatment prevented the destruction of islet structure (Fig 3A). Expression of Pdx1 and FoxO1, both of which are transcription factors critical for maintenance of beta cell mass and function [21, 22], was markedly decreased by alloxan treatment, but liraglutide clearly improved their expression (Fig 3A). We then quantified beta cell mass and found that the mass was ~20% of normal on the day following alloxan injection (day 0) and was gradually decreased in the vehicle-treated group to 10% on day 30 (Fig 3B). Liraglutide treatment prevented loss of beta cell mass during this period (Fig 3B). Alpha cell (glucagon-positive cell) mass was not changed by alloxan or liraglutide (Fig 3C).

### Liraglutide increases proliferation and decreases apoptosis

**Fig 4:**
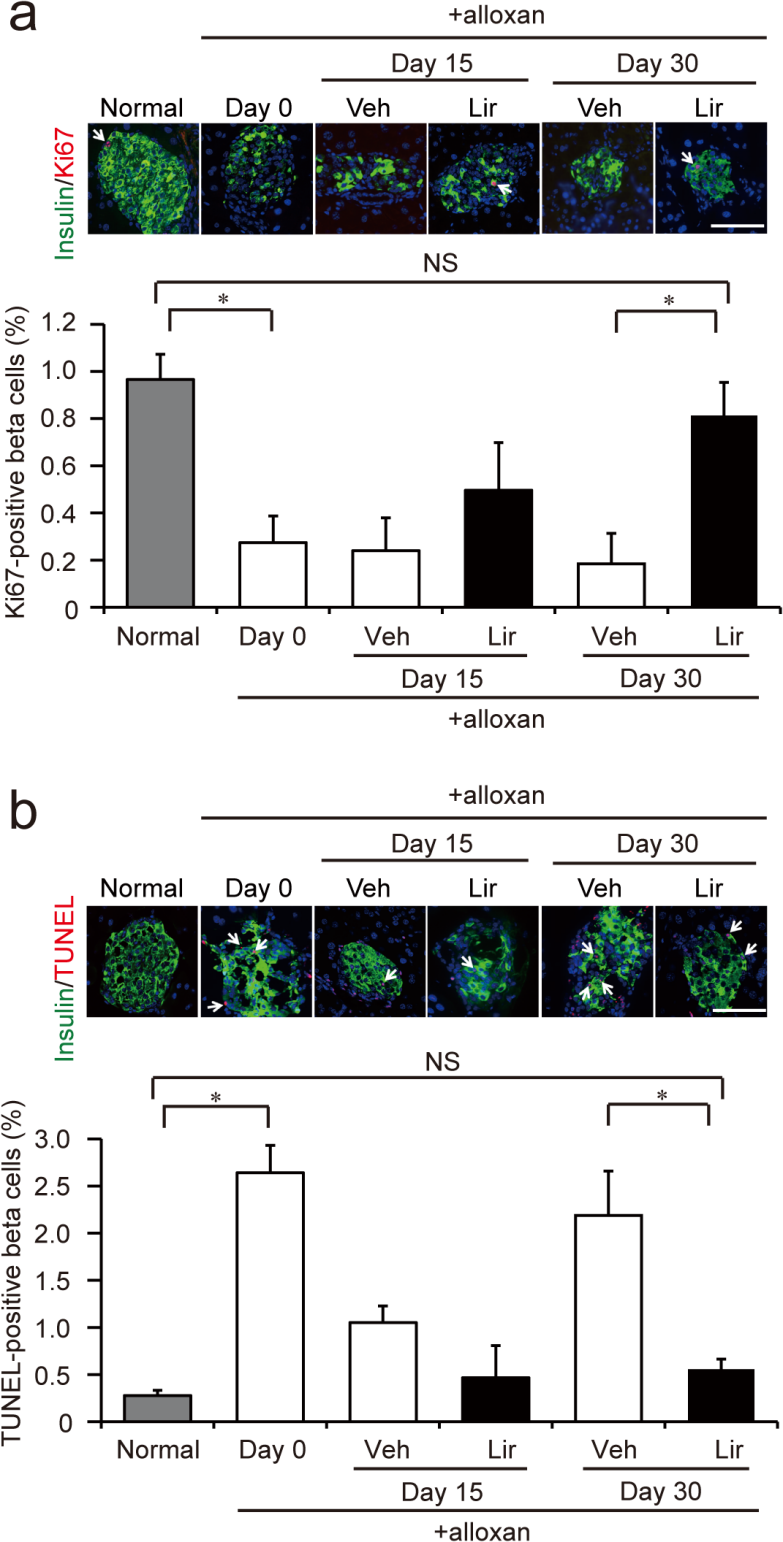
Proliferation and apoptosis of pancreatic beta cells. (**a**) Quantification of proliferating beta cells. Photographs are representative images of islets stained for insulin (green) and Ki67 (red). White arrows indicate Ki67- and insulin double-positive cells. Scale bars, 100 μm. The number of Ki67-positive beta cells was significantly increased in liraglutide-group compared to vehicle-group on day 30. (**b**) Quantification of apoptotic beta cells. Photographs are representative images of islets with insulin and TUNEL staining. White arrows indicate TUNEL- and insulin double-positive cells. Scale bars, 100 μm. The number of TUNEL-positive beta cells was significantly decreased in liraglutide-group compared to vehicle-group. White bars represent vehicle-treated group (Veh) (n = 4–6), and black bars represent liraglutide-treated group (Lir) (n = 4–6). NS, difference not significant. *p < 0.05

Proliferation of beta cells was evaluated by Ki67 staining. Ki67/insulin double-positive cells were approximately 1% of total insulin-positive cells in normal mice. Alloxan treatment significantly decreased the percentage to about 0.3%. Pancreatic beta cells of liraglutide-treated mice showed a significantly higher proliferating rate than that in vehicle-treated mice on day 30: the rate was comparable to that found in normal mice (Fig 4A). Apoptotic cell death of beta cells determined by the TUNEL method was significantly increased by alloxan treatment (~0.3% in normal to ~2.6% in alloxan-treated mice). Chronic liraglutide treatment decreased TUNEL-positive beta cells to a level similar to that in normal mice (Fig 4B). These data suggest that liraglutide prevents further loss of beta cells by both increasing the proliferation rate and decreasing apoptosis.

### Liraglutide does not induce beta cell neogenesis

**Fig 5:**
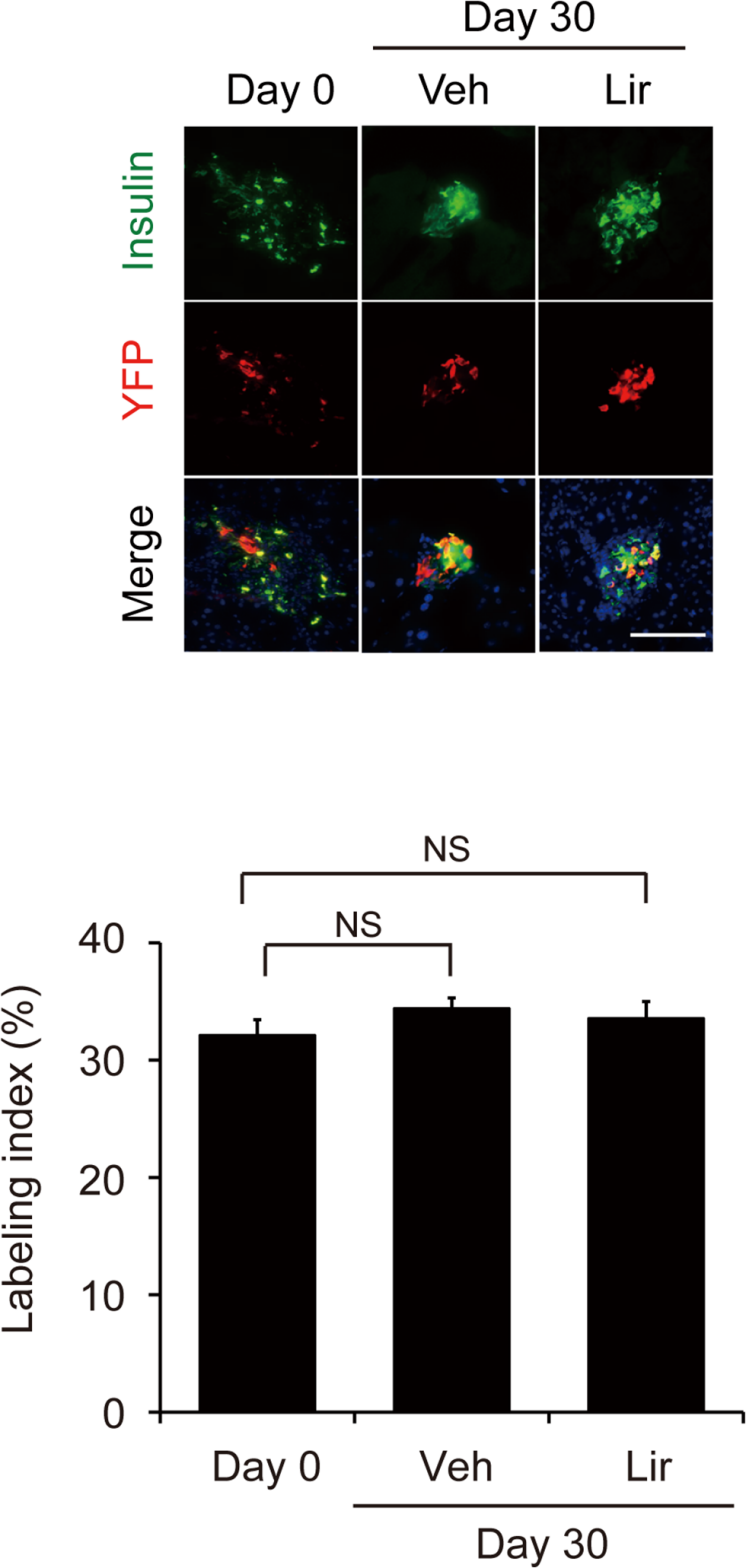
Evaluation of beta cell neogenesis. Double immunostaining for insulin (green) and YFP (red) indicates that pancreatic beta cells were specifically labeled by YFP. Scale bars, 100 μm. Quantification of YFP-labeled beta cells shows that the labeling index did not differ before and after liraglutide treatment. Veh, vehicle-treated group; Lir, liraglutide-treated group. Data are means ± SEM of five mice in each group. NS, difference not significant.

We next investigated whether liraglutide could induce neogenesis in alloxan diabetic mice by utilizing the inducible cell tracing system. Pancreatic beta cells of Ins2-CreER/R26R-YFP mice can be labeled at desired time points by injection of tamoxifen, and their progenies traced. If neogenesis occurs, the percentage of YFP-labeled beta cells (labeling index) should be decreased after the chase period. Indeed, we confirmed that the labeling index was decreased around weaning when neogenic islets appear [16, 23]. The labeling index before treatment of liraglutide (day 0) was 32.1%. The index was unchanged after 30-day liraglutide treatment (Fig 5), indicating that liraglutide does not induce beta cell neogenesis under conditions used in this study.

### Liraglutide suppresses oxidative stress

**Fig 6:**
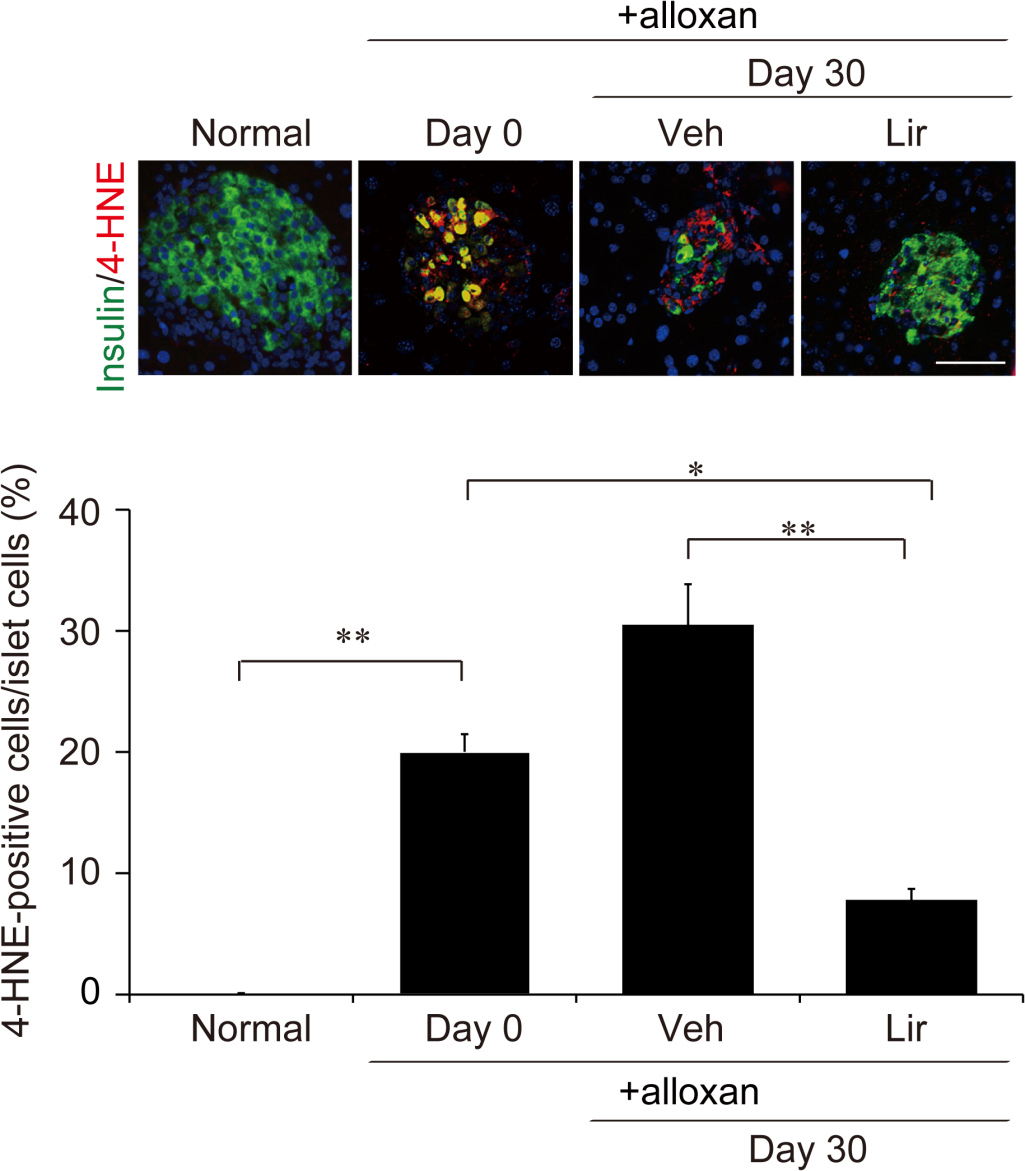
Oxidative stress assessed by 4-HNE staining. Photographs are representative images of islets stained for insulin (green) and 4-HNE (red). Scale bars, 100 μm. Quantification of 4-HNE-positive cells in islet cells is shown below the photographs. Alloxan treatment induced oxidative stress in pancreatic islets. Liraglutide treatment significantly decreased 4-HNE-positive cells in islet cells. Veh, vehicle-treated group; Lir, liraglutide-treated group. Data are means ± SEM of 4–5 mice in each group. *p < 0.05, **p < 0.01

It has been known that alloxan generates ROS and induces oxidative stress in pancreatic beta cells [17]. Chronic hyperglycemia also causes oxidative stress in beta cells, leading to impaired glucose-induced insulin secretion and apoptosis of beta cells [24]. We therefore examined whether liragutide treatment improves oxidative stress in alloxan diabetic mice using 4-HNE, a highly reactive diffusible product of lipid peroxidation, as a marker. Although 4-HNE immunoreactivity was barely detectable in pancreatic islets of normal mice, alloxan treatment markedly increased 4-HNE-positive cells (Fig 6). We found that liraglutide treatment significantly decreased the number of cells positive for 4-HNE in pancreatic islets (Fig 6). These results demonstrate that liraglutide suppresses oxidative stress in pancreatic beta cells of alloxan-induced diabetic mice.

### Improvements of beta cell mass and function as well as hyperglycemia are maintained after withdrawal of liraglutide treatment

**Fig 7:**
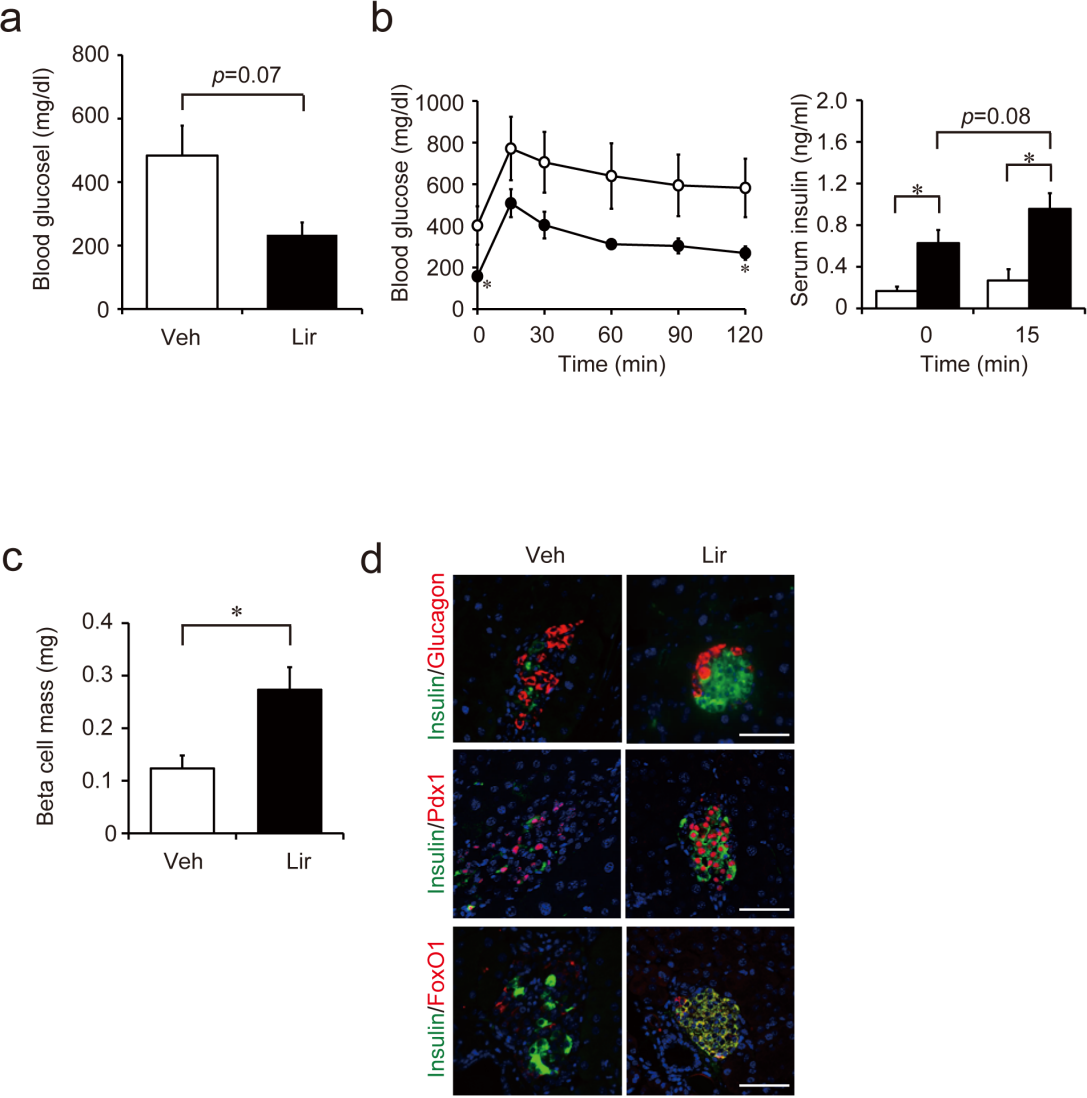
Beta cell function and mass two weeks after withdrawal of liraglutide treatment. (**a**) Casual blood glucose levels. Non-fasting blood glucose levels tended to be lower in liraglutide-treated group (n = 6) than in vehicle-treated group (n = 4). (**b**) Blood glucose levels (left) and serum insulin levels (right) during 1.5 g/kg OGTT. Liraglutide-treated group (n = 4) showed improved glucose tolerance as well as insulin response compared with vehicle-treated group (n = 3). (**c**) Beta cell mass. Beta cell mass was significantly higher in liraglutide-treated mice (n = 5) than in vehicle-treated mice (n = 3). (**d**) Insulin/glucagon (upper), insulin/Pdx1 (middle), and insulin/FoxO1 (bottom) double immunostaining. Representative images of islets are shown. Veh, vehicle-treated group; Lir, liraglutide-treated group. *p < 0.05.

Two weeks after the withdrawal of liraglutide treatment, blood glucose levels in the liraglutide-treated group still tended to be lower than those in the vehicle-treated group (Fig 7A). Moreover, glucose tolerance in liraglutide-treated mice remained improved with a concomitant increase in insulin secretion during OGTT (Fig 7B). The mass of pancreatic beta cells also was greater in the liraglutide-treated group than in the vehicle-treated group (Fig 7C). Furthermore, both Pdx1-positive cells and FoxO1-positive cells remained increased two weeks after stopping liraglutide administration (Fig 7D). Since the pharmacological half-life of liraglutide is about 13 hours in human and shorter in rodents [25], liraglutide should be washed out from the plasma of the mice two weeks after the end of administration. Thus, the effects of liraglutide on beta cell mass and function are sustainable after withdrawal of the drug.

## Discussion

Our data show that the human GLP-1 analogue liraglutide improves hyperglycemia concomitantly with increased beta cell mass and insulin secretion when administered daily for 30 days to alloxan-induced diabetic mice. We also found that these effects were maintained at least for two weeks after withdrawal of the liraglutide treatment.

Similar to our present findings, it has been reported that activation of GLP-1 receptor enhances proliferation and prevents apoptosis of beta cells [14]. Although pancreatic beta cell mass in adults is regulated primarily by the balance between proliferation and apoptosis of pre-existing beta cells, the involvement of new beta cells generated by neogenesis in the maintenance of beta cell mass is still unclear, mainly because methodology to ascertain the occurrence of neogenesis has not been established. The effect of GLP-1-related drugs on beta cell neogenesis in adult rodents has been evaluated based on the appearance of scattered small beta cell clusters and/or insulin-positive cells adjacent to ductal structures [26–32], both of which are indirect indications of newly differentiated beta cells. In the present study, we found that liraglutide did not increase the number of small beta cell clusters (S2 Fig), and we did not detect ductal insulin-expressing cells (S3 Fig). Moreover, the lineage tracing experiment indicated little or no contribution of beta cell neogenesis to the increase in beta cell mass following liraglutide treatment. We conclude that liraglutide increases proliferation and reduces apoptosis of the surviving beta cells after alloxan treatment but does not induce neogenesis under the conditions used. However, it cannot be ruled out that GLP-1 receptor agonists induce beta cell neogenesis in other conditions.

Although glucose plays a critical role in the physiological response of insulin secretion in pancreatic beta cells, chronic hyperglycemia causes impaired glucose-induced insulin secretion, altered gene expression, and apoptosis of beta cells [33]. These deleterious effects of chronically elevated blood glucose concentration have been referred to as glucotoxicity [34–36]. Conversely, it has been known that correction of hyperglycemia partially reverses beta cell defects [33]. In the present study, blood glucose levels in the liraglutide-treated group were significantly reduced compared to those in the vehicle group, but did not returned to normoglycemia: the levels were still around 400 mg/dl (Fig 1D). Accordingly, the beneficial effects of liraglutide cannot be attributed only to its glucose lowering effect.

Our results show that pancreatic islets of 30-day liraglutide-treated alloxan-induced diabetic mice are morphologically improved, concomitantly with improved expression of Pdx1 and FoxO1, resembling those in normal islet cells. These findings suggest that liraglutide may normalize beta cells damaged by alloxan and/or hyperglycemia. Indeed, the effects of liraglutide on beta cell mass and function were maintained even two weeks after drug withdrawal. Although little is known about sustainability of the effects of GLP-1 receptor agonists after drug withdrawal, recent clinical studies found that the beneficial effect was lost after the end of treatment of exenatide [11] and liraglutide [37] in type 2 diabetic patients. Species difference in proliferation/differentiation capacity of beta cells [38, 39], different regimens of the drugs, and/or different pathophysiological conditions may underlie this discrepancy.

One of the major mechanisms of beta cell defects induced by chronic hyperglycemia is oxidative stress. A high concentration of glucose increases ROS and oxidative stress markers in beta cells or islets of diabetic animals and human subjects [40–42]. Oxidative stress has been shown to inactivate beta cell transcription factors including Pdx1 and FoxO1, resulting in dysfunction of pancreatic beta cells [43]. Indeed, we found that the oxidative stress marker 4-HNE was increased and that expression of Pdx1 and FoxO1 was decreased in pancreatic islets of alloxan-treated mice. Liraglutide reduced oxidative stress, which may lead to the restoration of expression of these transcription factors and the improved beta cell function. It has been reported that exendin-4 decreases ROS production in diabetic GK rat islets through suppression of Src activation in an Epac-dependent manner [44]. As both liraglutide and exendin-4 activate the GLP-1 receptor and increase cellular cAMP levels, chronic treatment of liraglutide might reduce oxidative stress by suppressing Src activation. Thus, liraglutide could be effective in restoring oxidative stress-associated beta cell dysfunction, and increasing functional beta cell mass.

## Supporting Information

S1 Fig Functional impairment of beta cells by alloxan treatment. Blood glucose levels and serum insulin levels during 1.5 g/kg OGTT in alloxan-induced diabetic mice. Alloxan treatment severely impaired glucose tolerance and insulin response. Black circles and bars represent normal mice (n = 5), and white circles and bars represent alloxan-treated mice (n = 6). *p < 0.05, **p < 0.01.
(PDF)

S2 Fig Low magnification images of fluorescence photomicrograph of pancreas. Double immunostaining for insulin (green) and glucagon (red). Veh, vehicle-treated group; Lir, liraglutide-treated group. Scale bars, 1 mm.
(PDF)

S3 Fig No insulin-positive cells in ductal structures. Three different sections of pancreas of liraglutide-treated mice are shown. No insulin-positive cells (green) were detected in ductal structures (DBA-labeled cells, red). D, ductal structures. Scale bars, 100 μm.
(PDF)
