# Supplementary figures and images for: Liraglutide Improves Pancreatic Beta Cell Mass and Function in Alloxan-Induced Diabetic Mice
Source: PLoS One. 2015 May 4;10(5):e0126003. doi: 10.1371/journal.pone.0126003 (PMC4418765; doi:10.1371/journal.pone.0126003)

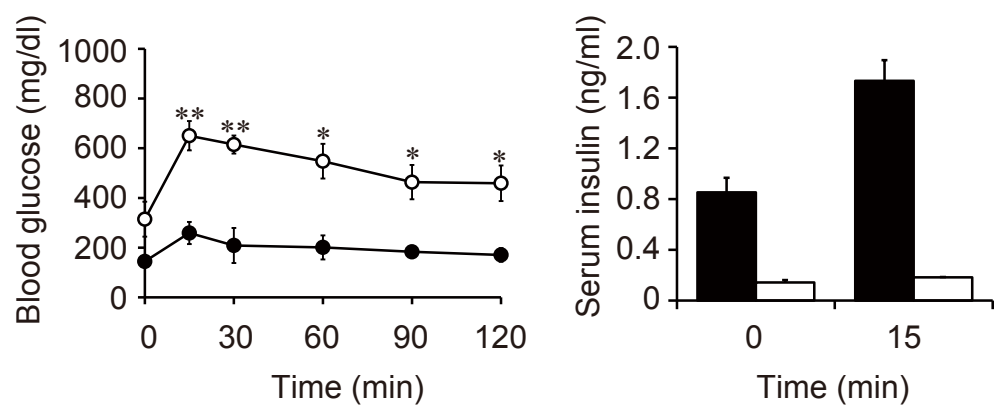

**S1 Fig. Functional impairment of beta cells by alloxan treatment.**

Supplement: S1 Fig — Blood glucose levels and serum insulin levels during 1.5 g/kg OGTT in alloxan-induced diabetic mice. Alloxan treatment severely impaired glucose tolerance and insulin response. Black circles and bars represent normal mice (n = 5), and white circles and bars represent alloxan-treated mice (n = 6). *p < 0.05, **p < 0.01. (PDF) [file pone.0126003.s001.pdf]

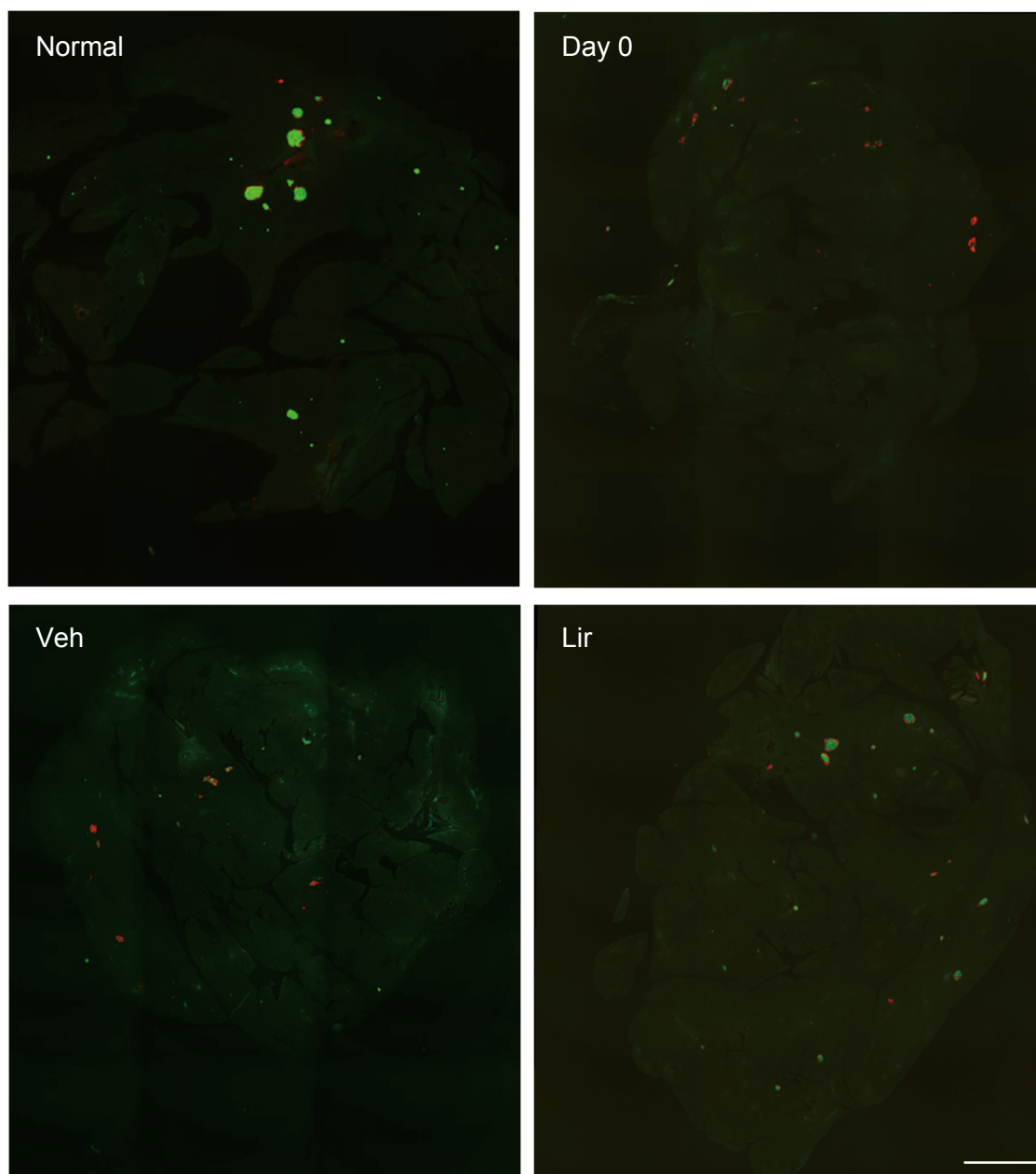

**S2 Fig. Low magnification images of fluorescence photomicrograph of pancreas.**

Supplement: S2 Fig — Double immunostaining for insulin (green) and glucagon (red). Veh, vehicle-treated group; Lir, liraglutide-treated group. Scale bars, 1 mm. (PDF) [file pone.0126003.s002.pdf]

Insulin/DBA/DAPI

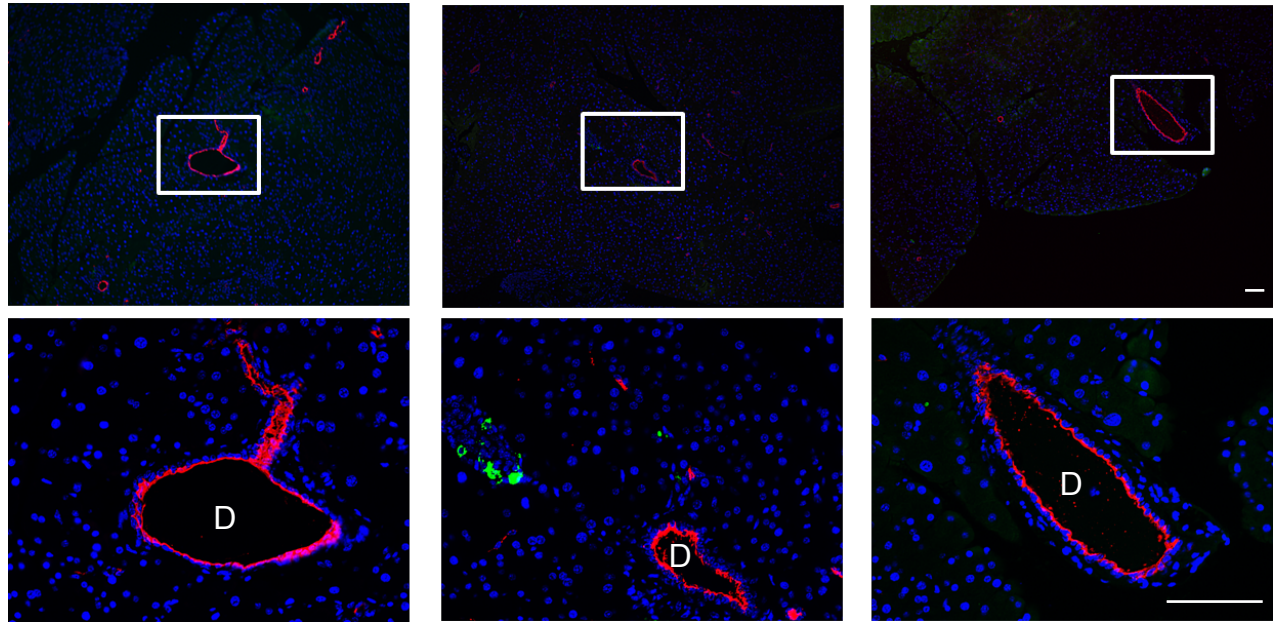

**S3 Fig. No insulin-positive cells in ductal structures.**

Supplement: S3 Fig — Three different sections of pancreas of liraglutide-treated mice are shown. No insulin-positive cells (green) were detected in ductal structures (DBA-labeled cells, red). D, ductal structures. Scale bars, 100 μm. (PDF) [file pone.0126003.s003.pdf]
